# The effect of fatty infiltration, revision surgery, and sex on lumbar multifidus passive mechanical properties

**DOI:** 10.1002/jsp2.1266

**Published:** 2023-07-17

**Authors:** Bahar Shahidi, Jennifer A. Padwal, Jeannie J. Su, Gilad Regev, Vinko Zlomislic, R. Todd Allen, Steven R. Garfin, Choll Kim, Richard L. Lieber, Samuel R. Ward

**Affiliations:** ^1^ Departments of Orthopaedic Surgery University of California and Veterans Administration Medical Centers San Diego California USA; ^2^ Departments of Radiology University of California and Veterans Administration Medical Centers San Diego California USA; ^3^ Departments of Bioengineering University of California and Veterans Administration Medical Centers San Diego California USA

**Keywords:** fat infiltration, multifidus muscle, paraspinal muscle, passive mechanics, spine, spine surgery

## Abstract

**Purpose:**

Previous research has demonstrated increased stiffness in the multifidus muscle compared to other paraspinal muscles at the fiber bundle level. We aimed to compare single fiber and fiber bundle passive mechanical properties of multifidus muscle: (1) in 40 patients undergoing primary versus revision surgery and (2) in muscle with mild versus severe fatty infiltration.

**Methods:**

The degree of muscle fatty infiltration was graded using the patients' spine magnetic resonance images. Average single fiber and fiber bundle passive mechanical properties across three tests were compared between primary (*N* = 30) and revision (*N* = 10) surgery status, between mild and severe fatty infiltration levels, between sexes, and with age from passive stress–strain tests of excised multifidus muscle intraoperative biopsies.

**Results:**

At the single fiber level, elastic modulus was unaffected by degree of fatty infiltration or surgery status. Female sex (*p* = 0.001) and younger age (*p* = 0.04) were associated with lower multifidus fiber elastic modulus. At the fiber bundle level, which includes connective tissue around fibers, severe fatty infiltration (*p* = 0.01) and younger age (*p* = 0.06) were associated with lower elastic modulus. Primary surgery also demonstrated a moderate, but non‐significant effect for lower elastic modulus (*p* = 0.10).

**Conclusions:**

Our results demonstrate that female sex is the primary driver for reduced single fiber elastic modulus of the multifidus, while severity of fatty infiltration is the primary driver for reduced elastic modulus at the level of the fiber bundle in individuals with lumbar spine pathology.

## Graphical abstract

Stiffness of the multifidus muscle may contribute to changes in load distribution and spinal stability. Our work found that female sex is the primary driver for reduced stiffness at the single fiber level, while muscle fatty infiltration is the primary driver for reduced stiffness at the fiber bundle level. This is the first study to investigate sex differences in muscle mechanics at the single fiber and fiber bundle level.

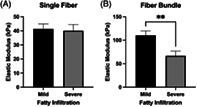

## 1 INTRODUCTION

The multifidus has distinct architectural features and performs over a range of sarcomere lengths^1^ that support its function as a posterior sagittal rotator of the lumbar vertebrae^2^ and stabilizer of the lumbar spine. Previous research demonstrated significantly higher passive mechanical properties of multifidus muscle fiber bundles compared to other paraspinal muscles.^3^ In this prior research, multifidus fiber bundles were 45% stiffer compared to other paraspinal muscles but were equivalent at the single‐fiber level. We found no correlation between single fiber and fiber bundle elastic modulus in the multifidus muscle, suggesting an extracellular origin to the muscle's unique passive mechanical properties. Although single fibers are composed of primarily the myofibrillar components (actin and myosin), fiber bundles also contain substantial extracellular matrix.

The significance of fatty infiltration in paraspinal muscle has been described in numerous studies, particularly by Parkkola and Kormano,^4^ who graded paraspinal muscle based on the severity of fatty infiltration with the assumption that adipose deposition in muscle was indicative of advanced pathology. Paraspinal muscles demonstrate an increase in fatty infiltration with age.^5^ However, multiple studies report a positive association between lumbar multifidus muscle fatty infiltration and low back pain in adult patients,^6^ and patients with unilateral radiculopathy or back pain have higher muscle atrophy^7^ and fatty infiltration^8^ in multifidus muscle on the symptomatic compared to asymptomatic side. Importantly, poor muscle quality has been shown to be predictive of post‐surgical failure in individuals undergoing spinal fusion surgery.^9^ In addition, changes in fatty infiltration in the presence of lumbar spine pathology appear to influenced by sex, with females demonstrating higher levels of fatty infiltration, as well as greater increases in fatty infiltration with age compared to men.^5^

Given the association between paraspinal muscle health and post‐surgical recovery, it is also important to consider the effect that lumbar spine surgery can have on posterior musculature. Post‐operative back pain, also known as “failed back syndrome” commonly occurs after spine surgery.^10^ Surgery‐induced trauma can lead to changes in muscle material properties and histology,^11^, ^12^ including acute enzymatic changes, muscle atrophy and fatty infiltration,^13^, ^14^ and reductions in postoperative muscle performance.^15^ Similarly, patients who have undergone multiple revision surgeries have greater functional impairments compared to those that have only had one surgery,^16^ and in an animal model of surgical injury, increased elastic modulus at the fiber bundle level has been observed.^17^ As such, minimally invasive surgical techniques that reduce disruption of the posterior paraspinal muscle compartment are becoming increasingly popular and, in many cases, demonstrate better clinical and radiographic outcomes compared to open approaches.^18^

The purpose of this study was to understand the passive mechanical properties of the multifidus muscle as a function of muscle health in patients undergoing lumbar spine surgery with: (1) mild versus severe fatty infiltration of the multifidus muscle and (2) primary versus revision surgery. Given the known influence of age and sex on atrophy and fatty infiltration in individuals with spine pathology, we also aimed to quantify the association between age and sex on these passive mechanical properties in the presence of fatty infiltration and revision surgery. We hypothesized that muscle fiber bundles from patients with severe fatty atrophy and patients undergoing revision surgery would have the highest stiffness.

## 2 MATERIALS AND METHODS

### 2.1 Subjects

After providing informed consent under a protocol approved by the local ethical review board, 40 patients who were undergoing spinal surgery for lumbar spine pathology including lumbar spine stenosis, disc degeneration or herniation, facet arthropathy, or spondylolisthesis were included in this study. For patients undergoing revision surgery, all patients were undergoing the second surgery, and the biopsy was taken within one level of the original (primary surgery) pathology. Multifidus biopsies were obtained from a standardized location (1 cm lateral to the spinolaminar border) at the level of pathology.^19^ Of these cases, data on fiber bundle characteristics for 23 of these participants have been included in a previous publication,^3^ but the influence of muscle quality, revision surgery, and sex was not examined at that time.

### 2.2 Specimen and preparation

A small segment of the multifidus muscle was identified and isolated by blunt dissection along natural fascicular planes. A specialized clamp was then slipped over the bundle with care to avoid undue manipulation or tension on the muscle.^1^ Small (~50 mg) biopsies of the multifidus muscle were obtained using an arthroscopic rongeur. After harvest, the biopsy was immediately placed in relaxing solution composed of (in millimoles per liter): ethylene–glycol tetraacetic acid (EGTA), 7.5; potassium propionate, 170; magnesium acetate, 2; imidazole, 5; creatine phosphate, 10; adenosine triphosphate (ATP), 4; leupeptin, a protease inhibitor, 17 mg/mL; and E64 (a protease inhibitor) 4 mg/mL.^20^ This solution prevented depolarization across any site of disrupted membrane and proteolytic degradation, either of which can destroy the specimen. Single fibers or fiber bundles were either immediately dissected from the fresh biopsy free of obvious external fat accumulation or placed into a storage solution composed of relaxing solution mixed with 50% glycerol and stored at −20°C. Samples stored in this manner have been shown to have stable mechanical properties for up to 3 months,^21^ but all fibers in this study were tested within 14 days of harvest.

### 2.3 Passive single‐fiber and fiber‐bundle mechanics

The single‐ and fiber‐bundle testing protocol was designed to measure elastic material properties apart from any velocity dependent properties, as previously described.^3^, ^22^ Briefly, the dissected fiber or fiber‐bundle segment was secured on either side to 125 μm titanium wires using 10‐0 silk suture loops. One wire was secured to an ultrasensitive force transducer (Model 405A, sensitivity 10 V/g, Aurora Scientific, Ontario, Canada) and the other was secured to a micromanipulator. The sample was transilluminated by a 7 mW He–Ne laser to permit sarcomere length measurement by laser diffraction.^23^ Resolution of this method is approximately 5 nm. The system was calibrated with a 2.50 μm plastic blazed diffraction grating prior to experimentation (Diffraction Gratings, Inc., Nashville, TN). After calibration and mounting, samples were lengthened until force registered on a load cell that defined baseline load and slack sarcomere length. Baseline sample diameters were optically measured with a cross‐hair reticule mounted on a dissecting microscope and micromanipulators on an x–y mobile stage. Force–displacement data were generated for each mounted sample in 250 μm increments after which stress‐relaxation was permitted for 2 min and both sarcomere length and tension were again recorded. Segments were elongated through the theoretical limit of actin and myosin overlap in human muscle. Force data were converted to stress by dividing force by the baseline cross‐sectional area value and displacement was converted to strain subtracting sarcomere length from the baseline slack sarcomere length value and then dividing by the baseline slack sarcomere length. The slope of the stress–strain curve between 2.0 and 4.25 μm was defined as the elastic modulus as previously described for human spine tissue.^1^, ^3^ Samples were discarded if they did not produce a clear diffraction pattern, if any irregularities appeared along their length, or if they were severed or slipped at either suture attachment point during the test.

### 2.4 Magnetic resonance imaging

Pre‐operative axial T1‐weighted magnetic resonance images at the level of the biopsy were reviewed from patient medical records and analyzed for fatty infiltration. Severity of fatty infiltration of the multifidus and erector spinae muscles together was qualitatively graded visually from clinical images by a single orthopedic surgeon rater as either mild (<25%) or severe (>25%). This threshold was chosen based on previously published grading systems recommended as described by Parkkola and Kormano.^4^

### 2.5 Data analysis

Three separate single‐fiber and fiber‐bundle passive mechanics experiments were averaged to obtain a single value per biopsy per patient. Independent *t*‐tests (for continuous variables) and chi‐square or Fisher exact tests (for categorical or binary variables) were performed to compare demographic and passive mechanical characteristics between those with mild versus severe fatty infiltration, and those undergoing primary versus revision surgery, and to evaluate independent variable distributions and intercorrelations. Cohen's d statistics were calculated for each comparison to generate effect sizes. Separate multivariable linear regressions were performed to quantify adjusted relationships between age, biological sex, degree of fatty infiltration, or revision status and dependent measurements of elastic modulus at the single fiber or fiber bundle level. Variables were checked for normality of variance using Levene's test for Equality of Variances and collinearity was checked using Variance Inflation Factor. All values are presented as mean ± standard error unless otherwise noted. Statistical tests were made using SPSS (version 28.0.1.1, Chicago, IL), with *p‐*values set to 0.05.

## 3 RESULTS

Of the 40 biopsy samples, fiber bundle mechanics were not successfully measured in one patient. Another patient was an extreme outlier for single fiber elastic modulus, with an average value measuring 179.7 kPa, which was more than 12 standard deviations from the group mean. This fiber modulus value was therefore excluded from the group analysis. As such, a total of 39 samples were available for passive mechanical analysis. Within‐subject coefficient of variation across the three experiments averaged 38% for the fiber bundles, and 20% for the single fiber analyses. Preoperative MR imaging was not available for six participants, and therefore fatty infiltration grading and comparison analyses were performed for a subgroup of 33 participants. Most participants were undergoing primary surgery (*N* = 30, 76.9%), and of the subgroup with fatty infiltration grading, most were categorized as mild (*N* = 23, 69.7%). Additionally, a small majority of participants were male (*N* = 20, 51.2%).

### 3.1 Fatty infiltration and surgery status

**FIGURE 1:**
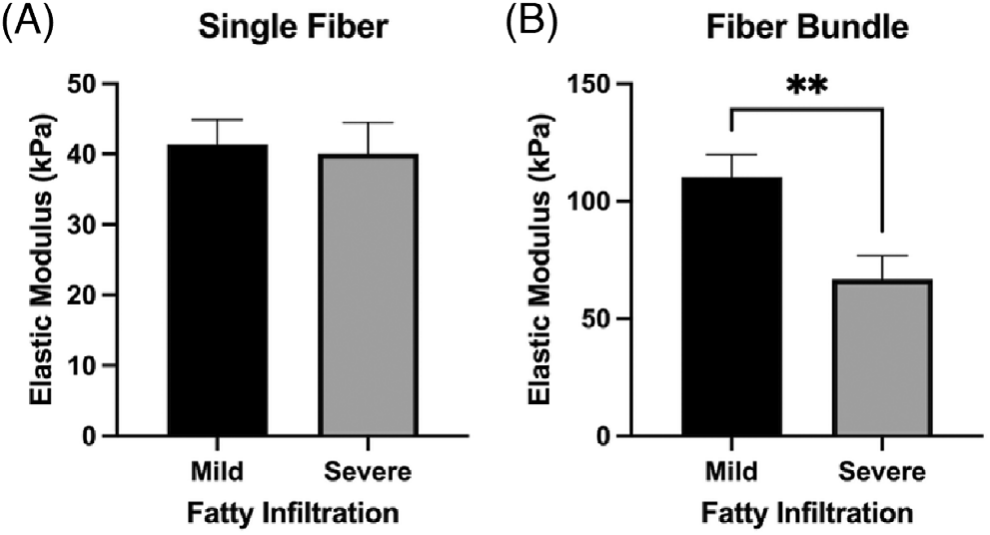
Comparison of (A) single fiber and and (B) fiber bundle elastic modulus (EM) in the multifidus muscle by degree of fatty infiltration. Data are presented as mean ± SEM. **indicates *p*‐value < 0.05.

**FIGURE 2:**
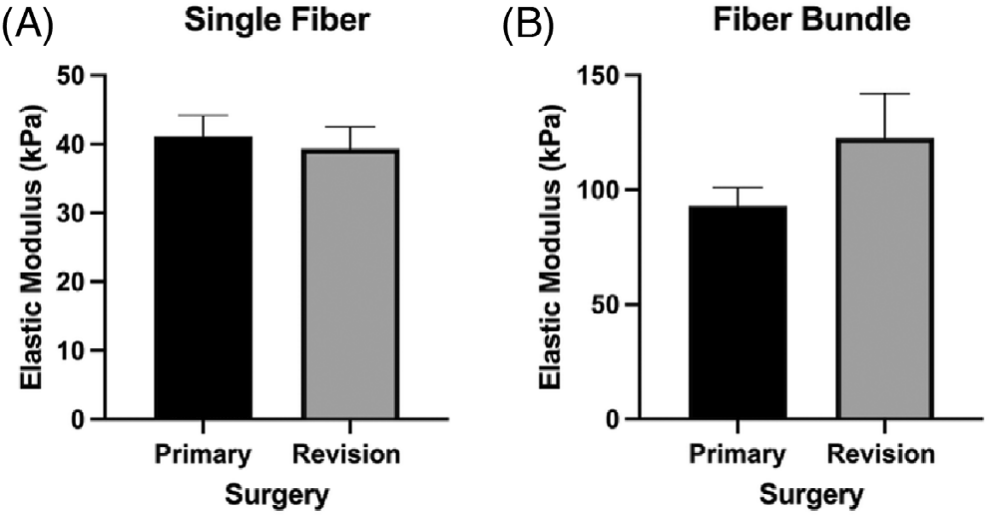
Comparison of (A) single fiber and (B) fiber bundle elastic modulus (EM) in the multifidus muscle by surgery status. Data are presented as mean ± SEM.

From the univariate comparisons, patients with mild fatty infiltration trended toward being younger (Cohen's *d* = −0.74, *p* = 0.06), and had a lower body mass index (BMI, Cohen's *d* = −1.04, *p* = 0.02) as compared those with severe fatty infiltration (Table 1). There was no difference in single fiber elastic modulus between patients with mild versus severe fatty infiltration (Cohen's *d* = 0.08, *p* = 0.84, Figure 1A). At the fiber bundle level, elastic modulus was reduced in patients with severe fatty infiltration (66.73 ± 10.15 kPa) compared to patients with mild fatty infiltration (110.3 ± 9.42 kPa) by 40% (Cohen's *d* = 1.1, *p* = 0.01) (Table 2, Figure 1B). There were no significant differences in demographic or passive mechanical characteristics between patients undergoing primary versus revision surgery; however, the fiber bundles were observed to be approximately 30% stiffer in revision cases as compared to primary cases, which, although not significant, demonstrated a medium effect size (Cohen's *d* = −0.64, *p* = 0.10), (Tables 1 and 2; Figure 2A,B).

**TABLE 1:** Patient demographics of stratified by fatty atrophy and surgery status.

	Fatty atrophy			Surgery		
	Mild	Severe	*p*‐value	Primary	Revision	*p*‐value
Age (years)	57.8 ± 2.4	66.4 ± 3.3	0.06	58.8 ± 2.4	64.3 ± 5.7	0.30
BMI	26.0 ± 1.1	31.4 ± 2.1	**0.02**	27.3 ± 1.1	27.1 ± 2.2	0.91
% Female	47.8	52.2	0.91	48.7	51.3	0.64
Biopsy level (*n*)						
L2‐3	1	1		2	0	
L3‐4	3	1		2	3	
L4‐5	13	7		19	5	
L5‐S1	6	1		7	1	

*Note*: Data presented as mean ± SEM.

Abbreviation: BMI, body mass index.

**TABLE 2:** Biomechanical testing of single fiber and fiber bundles.

	Mild	Severe	*p*‐value	Primary	Revision	*p*‐value
Diameter (mm)						
Single fiber	0.109 ± 0.004	0.108 ± 0.007	0.85	0.108 ± 0.004	0.111 ± 0.005	0.76
Fiber bundle	0.299 ± 0.017	0.345 ± 0.022	0.12	0.314 ± 0.018	0.317 ± 0.013	0.92
Sarcomere slack length (μm)						
Single fiber	2.11 ± 0.04	2.19 ± 0.09	0.38	2.12 ± 0.04	2.09 ± 0.09	0.72
Fiber bundle	2.08 ± 0.04	2.17 ± 0.06	0.18	2.09 ± 0.03	2.12 ± 0.07	0.67
Elastic modulus (kPa)						
Single fiber	41.34 ± 3.55	40.06 ± 4.43	0.84	41.16 ± 2.98	39.35 ± 3.20	0.76
Fiber bundle	110.35 ± 9.42	66.73 ± 10.15	0.009	92.90 ± 7.97	122.67 ± 19.37	0.10

*Note*: Values are mean ± SEM.

### 3.2 Age and biological sex

**FIGURE 3:**
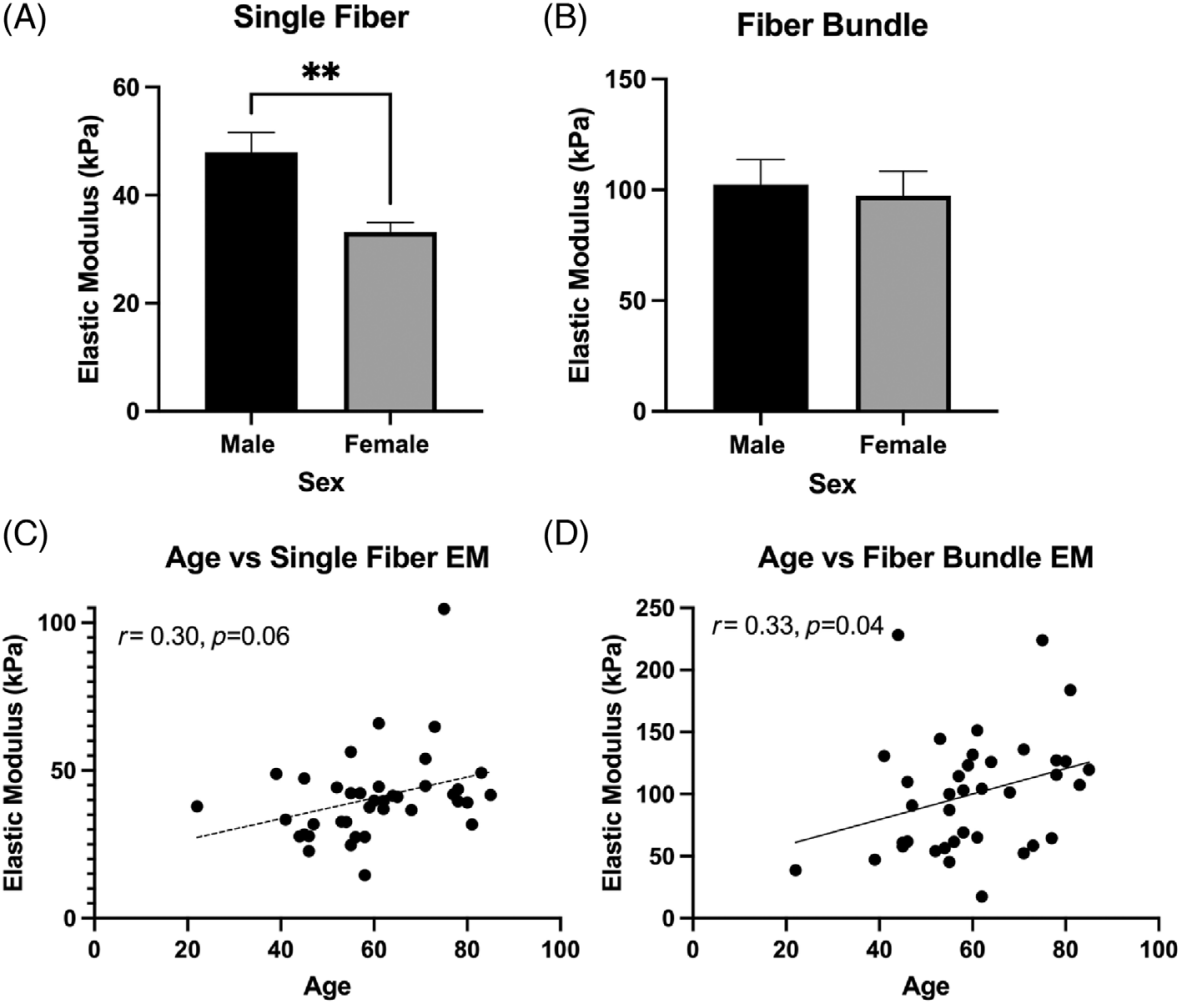
Comparison of (A) single fiber and (B) fiber bundle elastic moduli (EM) in the multifidus muscle by sex. Women had significantly reduced single fiber EM compared to men. The bottom row represents scatter plots of age versus EM for single fiber (C) and fiber bundle (D) data. Data are presented as mean ± SEM. ** indicates significant differences (*p* = 0.001). Solid linear regression fit line indicates a significant association, while a dotted fit line indicates a trend (*p* < 0.1).

Females demonstrated significantly reduced stiffness at the single fiber level (Cohen's *d* = 1.1, *p* = 0.001) (Figure 3A). At the fiber bundle level, there was no association between biological sex and elastic modulus (Cohen's *d* = 0.10, *p* = 0.75, Figure 3B). There was a trend toward increasing stiffness with older age (*r* = 0.30, *p* = 0.06; Figure 3C) at the fiber bundle level, and a significant association with older age and stiffness at the single fiber level (*r* = 0.33 *p* = 0.04; Figure 3D). The observed associations were retained in the adjusted multivariate model, with the association between age and fiber bundle elastic modulus becoming significant (*p* = 0.048, Table 3).

**TABLE 3:** Multivariable determinants of elastic modulus for single fiber and fiber bundle groups.

	Single fiber		Fiber bundle	
	B coefficient (SE)	*p*‐value	B coefficient (SE)	*p*‐value
Age (years)	0.52 (0.21)	0.019	2.25 (0.51)	<0.001
Biological sex (male reference)	−15.7 (4.7)	0.002	−16.0 (11.3)	0.168
Fat infiltration (mild reference)	−5.5 (5.3)	0.316	−63.9 (13.0)	<0.001
Surgery status (primary reference)	−2.3 (5.9)	0.692	−2.2 (14.1)	0.876

## 4 DISCUSSION

The primary purpose of this paper was to compare passive mechanical properties of the multifidus muscle across surgical states and levels of fatty infiltration. The secondary purpose was to evaluate the impact of age and biological sex on these properties in individuals with lumbar spine pathology. We hypothesized that muscle fiber bundles from patients with severe fatty infiltration and from patients undergoing revision surgery would be stiffer based upon (1) our assumption that fibrosis would be higher in revision surgery, and (2) previous literature suggesting that connective tissue/extracellular matrix dominates fiber bundle properties, and is present in greater proportions in tissue with high levels of fatty infiltration.^3^, ^19^, ^24^, ^25^ Our results were contradictory to our hypothesis in that we found reduced bundle stiffness in individuals with severe fatty infiltration and younger age. Although we found no statistically significant impact of revision surgery at either bundle or single fiber levels, the elastic modulus was approximately 30% higher with a moderate effect size at the fiber bundle level in individuals undergoing revision surgery. Interestingly, our findings demonstrated that the primary drivers for single fiber mechanical properties were age and sex, with sex explaining 23% of the variance in single fiber elastic modulus and age explaining 8.5% of the variance. These data suggest that there may be different passive mechanical properties between males and females in human patients with lumbar spine pathology that warrant further studies. These data represent novel evidence of sex‐specific mechanical properties of muscle at the single muscle fiber level.

### 4.1 Passive mechanical properties in pathological muscle

The architectural features of the lumbar stabilizing muscles provide resistance to internal and external loads, and passive mechanical properties have additional implications for mechanical function.^26^ Variability in passive mechanical properties across these muscles supports the role of the multifidus as a key stabilizer in resisting flexion moments.^1^, ^3^ Despite data supporting the functional relevance of passive mechanical properties of the lumbar multifidus in stabilization of the spine, there are very few studies assessing elastic modulus directly from humans with pathological conditions of varying severities. Current studies demonstrate increases in muscle fiber stiffness in human patients with spasticity associated with cerebral palsy,^27^ human rotator cuff tears,^28^ and after botulinum toxin injections in animal, but not human models.^29^

Literature in spine‐specific pathologies is even more limited. In an animal model of intervertebral disc degeneration, both multifidus muscle fibers and fiber bundles were stiffer 12 weeks after injury,^30^ although in a model of spinal stiffness induced by ectopic calcification in ENT1‐deficient mice, reduced single fiber stiffness was observed, with no change in fiber bundle mechanics.^31^ Interestingly, a recent study investigating passive mechanical properties of the multifidus muscle in individuals with adult spinal deformity demonstrated extremely high fiber bundle elastic modulus in some patients, resulting in increased simulated spinal compressive loads of over 500%.^32^ Similar to our own data, this study reported histopathological findings of fibro‐fatty replacement and muscle degeneration in these patients. The lower elastic modulus in individuals with severe fatty infiltration is, perhaps, not surprising given that adipocytes often accumulate in the interstitium, although our biopsy samples were carefully cleaned of extramuscular fatty tissue prior to passive mechanical testing. The impact of these passive mechanical changes in the presence of severe muscle fatty infiltration on clinical function is not well understood. However, it is important that these changes be considered in biomechanical assessments of normal or pathological spine.

Previous literature regarding the passive mechanical properties of the multifidus muscle in individuals undergoing surgery is conflicting. Our own previous work demonstrated increased stiffness after experimentally induced disc degeneration in a rabbit model,^30^ and previous animal studies on muscle injury induced from surgical trauma have demonstrated increased stiffness of the multifidus.^17^ However, another study in an animal model of facet and fascial injury demonstrated no differences in multifidus passive mechanical properties 28 days post injury.^33^ Given the observations previously described related to associations between muscle fatty infiltration and stiffness, these conflicting results may be due to the presence of additional confounders in a human sample including variable etiology, pathological severity, presence of muscle degeneration, and aging.

### 4.2 The influence of biological sex and age on muscle passive mechanics

Contrary to our previous study,^3^ we found an association between age and elastic modulus at the fiber bundle level along with sex and elastic modulus at the single fiber level. This is consistent with previous literature in other muscles demonstrating an age‐related increase in muscle fiber stiffness,^34^, ^35^ although some studies caution that these relationships are modulated by muscle length^36^ and joint position.^37^ Unlike aging, sex‐specific differences in passive mechanical properties have not been well elucidated. In ultrasound elastography studies of passive whole muscle stiffness, results are conflicting with both increased,^38^, ^39^ decreased,^40^, ^41^ or no difference^42^ in stiffness for females compared to males. Surprisingly, although active mechanics of muscle have been investigated, no studies, to our knowledge, have compared passive mechanical properties of single fiber and fiber bundles between human males and females. The lower elastic modulus at the single fiber level in female patients could represent differences in intracellular components such as titin,^43^ which appear to be counterbalanced at the fiber bundle level by reduced stiffness of the extracellular matrix. Alternatively hormonal factors such as estrogen level may also play a role in skeletal muscle elasticity. Indeed, some evidence of estrogen‐driven muscle fibrosis has been observed in abdominal muscles in a mouse model of inguinal hernia.^44^ Future research is needed to further investigate differences in passive mechanical properties between males and females.

Several limitations exist in this study. Mainly, our samples were obtained from patients undergoing surgery and the associations we observed could be attributed to the pathological nature of the tissue. Future research is needed to compare these results to individuals with healthy paraspinal muscle. Second, we had a limited number of samples of revision cases (nine samples), which may have limited the ability to power comparisons across surgery states. We did observe a moderate effect size and a low, but non‐significant *p*‐value, which suggests that these observations have merit for further investigation. Finally, our fiber and fiber bundle cross‐sectional area calculations were based on sample diameter, which requires the assumption that the structures are circular. Although there is historical precedent for this approach, recent data suggests that this assumption may not be valid and could lead to errors in stress calculations.^45^

## 5 CONCLUSION

Our data are the first to investigate sex‐specific differences in passive mechanical properties of muscle in humans, with females demonstrating significantly reduced multifidus elastic modulus at the single fiber, but not fiber bundle level. We found that the mechanical properties of the multifidus muscle fiber bundle in the presence of lumbar spine pathology change by approximately 40% depending on the disease state, and revision surgery may have an influence on muscle stiffness at the fiber bundle level. Finally, we confirmed previous findings that increased age is associated with increased muscle elastic modulus. These observations are likely to be important modifiers of lumbar spine stability and may contribute to pathokinesiology in patients with lumbar spine disease.

## FUNDING INFORMATION

This work was supported by the following grants: 1TL1RR03197, R01HD088437, 2R24HD050837, and 5TL1TR001443‐02.

## CONFLICT OF INTEREST STATEMENT

The authors declare no conflicts of interest.
